# Pneumococcal vaccination in children with underlying conditions

**DOI:** 10.1186/1824-7288-41-S2-A59

**Published:** 2015-09-30

**Authors:** Nicola Principi, Susanna Esposito

**Affiliations:** 1 Pediatric Highly Intensive Care Unit, Department of Pathophysiology and Transplantation, Università degli Studi di Milano, Fondazione IRCCS Ca’ Granda Ospedale Maggiore Policlinico, Milan, Italy

## 

Several epidemiological studies have evidenced that children with underlying chronic conditions are at increased risk of pneumococcal infections with higher case-fatality rates than healthy subjects. Because pharyngeal pneumococcal carriage is a prerequisite for pneumococcal infection development, the high colonization rates found in these subjects is the most important reason for this phenomenon [1]. To overcome this problem, health authorities worldwide have recommended that children with severe chronic disease receive pneumococcal vaccine prophylaxis with the 13-valent conjugate preparation (PCV13) not only in the first years of life but also during school age and adolescence. Conjugate pneumococcal vaccines significantly reduce pneumococcal carriage of serotypes included in the vaccines and consequently limit development of pneumococcal infections. A dose of PCV13 has been added to the 23-valent polysaccharide vaccine in order to obtain a stronger protection at least against the 13 pneumococcal serotypes included in this vaccine. The choice of PCV13, already found extremely effective in reducing pneumococcal infections and pharyngeal carriage rates in both vaccinated healthy children and unvaccinated healthy individuals, was based on a large number of studies that have clearly evidenced that this vaccine can evoke a protective immune response in most of the subjects with underlying disease with a satisfactory profile of tolerability and safety [2].

Unfortunately, despite recommendations, pneumococcal vaccination coverage remains in most of the children at increased risk of pneumococcal complications significantly lower than desired. Limited knowledge about the real importance of pneumococcal infections in children at increased risk by patients, parents and physicians themselves together with a not justified fear of poor safety and tolerability of the vaccine are the most important reasons for the low compliance with official recommendations [3]. Specific educational programs have to be planned if this problem has to be solved.

However, the use of a single dose of PCV13 could not definitively solve the problem of the protection of children at risk from pneumococcal infection. The duration of protection offered by this vaccine is not established and it is not defined whether one or more booster doses have to be given. Moreover, with time it is possible that a new replacement phenomenon could take place and this could require the use of a vaccine able to cover a greater number of serotypes.
